# Reliabilities of Mental Rotation Tasks: Limits to the Assessment of Individual Differences

**DOI:** 10.1155/2013/340568

**Published:** 2013-09-30

**Authors:** Gerrit Hirschfeld, Meinald T. Thielsch, Boris Zernikow

**Affiliations:** ^1^German Paediatric Pain Centre, Children's Hospital Datteln, Dr. Friedrich-Steiner Street 5, 45711 Datteln, Germany; ^2^Department of Children's Pain Therapy and Paediatric Palliative Medicine, Witten/Herdecke University, Alfred Herrhausen-Straße 50, 58448 Witten, Germany; ^3^Westfälische Wilhelms-Universität Münster, Fliednerstr. 21, 48149 Münster, Germany

## Abstract

Mental rotation tasks with objects and body parts as targets are widely used in cognitive neuropsychology. Even though these tasks are well established to study between-groups differences, the reliability on an individual level is largely unknown. We present a systematic study on the internal consistency and test-retest reliability of individual differences in mental rotation tasks comparing different target types and orders of presentations. In total *n* = 99 participants (*n* = 63 for the retest) completed the mental rotation tasks with hands, feet, faces, and cars as targets. Different target types were presented in either randomly mixed blocks or blocks of homogeneous targets. Across all target types, the consistency (split-half reliability) and stability (test-retest reliabilities) were good or acceptable both for intercepts and slopes. At the level of individual targets, only intercepts showed acceptable reliabilities. Blocked presentations resulted in significantly faster and numerically more consistent and stable responses. Mental rotation tasks—especially in blocked variants—can be used to reliably assess individual differences in global processing speed. However, the assessment of the theoretically important slope parameter for individual targets requires further adaptations to mental rotation tests.

## 1. Introduction

Most cognitive studies are aimed at describing and explaining mean differences between groups of participants or experimental conditions. Within these studies, differences between participants belonging to the same experimental group are minimized as much as possible. For example, in a study that is trying to establish the effect of an intervention on mental rotation tasks, differences within the experimental and the control group should be minimal [1]. In contrast to this approach, recent studies used the variation between participants to approach substantial research questions about the processes underlying mental rotation in healthy adults [2] and patients [3]. However, if mental rotation tasks are used to characterize individuals, it becomes highly relevant to ensure that these tasks have an adequate reliability, as reliability provides an upper limit for the correlation to other variables. For example, the reported nonsignificant correlation between the mental rotation task and disease parameters [3] may be due to low internal consistencies of mental rotation tasks. Research into the implicit association task [4] and the dot-probe task [5] has shown that these implicit tasks suffer from very low internal consistencies and test-retest reliabilities. Comparable analysis for mental rotation tasks has only been done for letters and numbers [2] but not for objects or body parts. In the present study, we systematically investigate whether and how detailed researchers can assess individual differences using a specific variants of mental rotation tasks in which body parts are used as targets, for example, the hand laterality task (HLT).

The HLT is a variant of the mental rotation tasks in which participants are presented with pictures of (rotated) left or right limbs and have to decide whether a left or right limb is shown [6, 7]. What makes this task so interesting from a cognitive neuroscience perspective is that participants access the same mental representations to solve these tasks that are also used in planning everyday actions. In line with this, chronic pain patients are slower to identify limbs depicted in postures that they find difficult to attain [8, 9]. It is known for some time that motor imagery processes can be dissociated from visual imagery processes by contrasting nonbody and body targets [10, 11] (see [12] for evidence of the activation of motor activation in mental object rotation). Using not only hands but also feet [13] and faces [3] as targets enables a highly detailed characterization of the changes in body perception during the course of a pain disease. Furthermore, testing hypothesis about correlations between disease parameters such as severity or duration and mental rotation performance is only possible if both can be assessed with sufficient reliability.

Models of mental rotation tasks assume that performance can be partitioned into subprocesses that are related to mental rotation proper and processes related to peripheral processes, such as visual encoding, response generation, and decision making [2]. Mental rotation proper is believed to be dependent on the angle of rotation at which the targets are presented; that is, the longer it takes to respond to a rotated target the larger the angle of rotation is. While models of how the visual system achieves object constancy offer alternative explanation, that does not involve mental rotation, the mostwidely used variant where participants have to discriminate mirror-inverted pictures that are rotated in the picture plane is believed to involve mental rotation [14]. In line with a mental rotation explanation, a linear regression of response time on angle of rotation can be used to differentiate the two. In such analysis, the intercept of the regression represents the efficiency of the peripheral processes, while the slope represents a measure of the speed of mental rotation proper [15]. That is, exceptionally fast muscle conductance would affect all angles of rotation similarly and lead to faster overall reaction times, reflected by a small intercept of the regression equation. In contrast, if a particular participant has a very high rate of mental rotation, reaction times will only slightly increase with the increasing of the angle of rotation, reflected by a small slope of the regression equation.

Thus, the aim of the present research was to test whether the mental rotation task can be used to assess individual differences and how detailed different subprocesses involved in mental rotation can be studied. Based on the studies of the reliability of implicit personality tests [5], we surmised that even measures derived from reaction time experiments should (a) be very similar for two halves of the same test and (b) be stable over time. We included four different target types to assess both visual imagery (for cars) and motor imagery (for hands, feet, and faces) that were used in a previous research with healthy participants [6] and patients [3]. In order to test how different target types could best be combined into a single paradigm, we also compared a blocked and a mixed variant of the mental rotation task.

## 2. Materials and Methods

### 2.1. Participants

In total 99 healthy students (79 females; mean age 21.2 ± 3.4 years) from the University of Münster voluntarily took part in the mental rotation experiment. All were native speakers of German and had normal or corrected to normal vision. No participant reported any history of neurological or psychiatric disorders. They all gave informed consent before the experiment as laid out by the Declaration of Helsinki. Data was collected, stored, and analyzed anonymously. Participants received course credits for their participation.

### 2.2. Mental Rotation Experiment

**Figure 1:**
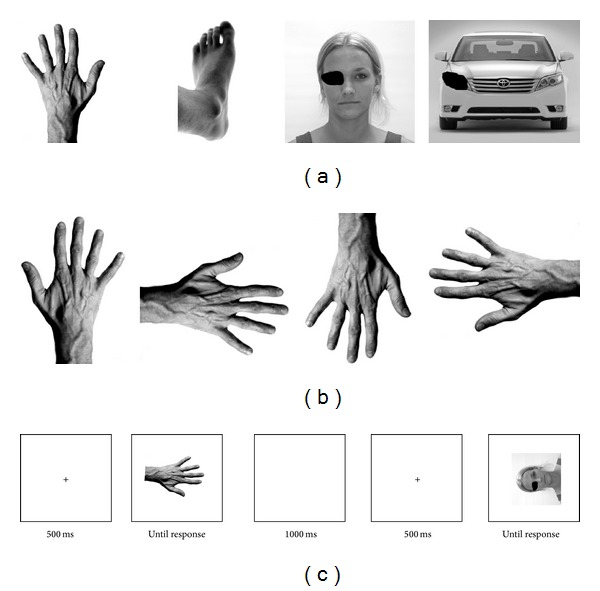
Experimental design. (a) Stimuli used in the experiment (left version of stimuli). (b) Angles of rotation (right hand as an example). (c) Trial timing.

In the mental rotation experiment, four different photographs (black and white, 72 dpi) were presented as targets for a left-right decision: (1) a photograph of a hand in a palm-down view, (2) a photograph of a foot, (3) a photograph of a face of a woman with one eye covered by a black spot and (4) a photograph of a car with one headlight covered by a black spot (Figure 1(a)). The picture of the females face was taken from a standardized database [16]. The other stimuli were taken from sources on the Internet, for example, Wikimedia Commons and can be solicited from the first author.

Participants had to indicate whether the picture depicted a left or a right version of the stimulus. The two versions were generated by mirroring the image along the horizontal picture plane. Before the start of the experiment, participants were given instructions that showed both versions of the four targets together with the correct response button. This was necessary as the correct response for the face and car would have been ambiguous otherwise. Each version was presented at four rotational angles (0, 90 lateral, 90 medial, or 180; Figure 1(b)), resulting in 32 trials per target type and 128 trials per participant. There were three short breaks after every 32 trials. Each trial began with the presentation of a fixation cross in the middle of the screen for 500 ms, followed by the target picture. Target pictures were presented until a response was given, followed by a blank screen presented for 1000 ms (Figure 1(c)).

Participants were randomly assigned to one of two groups that differed in how the four different target types were presented. In the “blocked” group the different target types were presented in homogeneous blocks consisting of only hands, only feet, only faces, or only cars. In the “mixed” group the different targets were presented in random order. Within each block the order was randomized separately for each participant. In the blocked group the order of target types was balanced between participants using a Latin square design. The overall number of stimulus presentations and breaks was similar in the two groups. The experiment took about 15 minutes to complete.

Participants were invited to repeat the experiment approximately six weeks after the experiment. 63 (64% of original sample) students (50 females; mean age 21.7 ± 3.5 years) took part in this retest after on average 45.3 ± 8.7 days.

### 2.3. Data Analysis

Raw data were screened for erroneous responses (7.8%) and responses faster than 100 ms or slower than 1750 ms (8.7%). Overall, we found no speed for accuracy tradeoff. Instead, correct responses were given faster than incorrect responses (*P* < .001). As similar pattern of results arose for error rates, we will focus on reaction time data in the following.

In order to show that the overall participants responded as expected, a group-level analysis was performed using linear mixed effect models [17]. These analyses replace traditional ANOVA approach that entail averaging responses over similar conditions as they allow modeling response times for each individual correct response using both fixed and random effects. In our analysis trial number (*1 to 32 *within each block), gender, orientation (0, 90, and 180 degrees), target type (hand versus feet, and versus face versus car), and variant (mixed versus blocked) were used as fixed effects and random intercepts for each participant and target type separately. There are some debates about whether or not nested random effects should be included. The more complex random effect structure was chosen to resemble more closely the analysis at the individual level and to improve the validity of the inference [18].

The main analysis concerned the consistency (split-half reliability) and stability (test-retest reliability) of the intercepts and slopes computed for each individual. The split-half reliability was computed in four steps. First, the responses of each participant were randomly split into two halves (“a” and “b”). Second, for each participant two intercepts and slopes were estimated using the responses from halves “a” and “b.” Intercepts and slopes were estimated by fitting linear models with orientation (0, 90, and 180 degrees) and trial number (*1 to 32* within each block) to the response times for every participant, first for all trials and then for the four-target types separately. In keeping with the group-level analysis, no trials were averaged before fitting these models. This resulted in five different models—each yielding an intercept and a slope—for every participant and half. Third, the correlation coefficient between the slopes and intercepts from the two halves was computed to estimate the magnitude and the significance of the correlation. Fourth, the Spearman-Brown formula (*r*_Spearman-Brown_ = (2∗*r*_measured_)/(1 + *r*)) was used to predict the reliability of the intercept and slope estimates if these were estimated from all trials. This is necessary as the model parameters estimated from half of the trials have lower reliability than those estimated from the full set.

The test-retest reliability was computed using the same three steps as described above quantified by computing the correlation between the intercepts and slopes estimated from the two measurements six weeks apart. Reliabilities were interpreted according to commonly accepted standards [19].

## 3. Results

### 3.1. Group-Level Analysis

At the group-level analysis we found that response time was affected by several factors above and beyond the random factors (Figure 1). There were main effects for trial number (beta = −3.01; std = .25; *t* = −12.15; *P* < .001), rotation (beta = 2.12; std = .13; *t* = 16.61; *P* < .001), variant (beta = 99.05; std = 33.34; *t* = 2.97; *P* < .001), and hand-targets (beta = 163.50; std = 32.97; *t* = 4.96; *P* < .001), indicating slower responses for the first items of each block, target at large angles of rotation, the mixed presentation condition, and hands. Furthermore, there was a significant interaction between foot targets and rotation (beta = .48; std = .18; *t* = 2.63; *P* < .01), indicating a larger rotation-effect for feet. Importantly, all other two-way and higher-order interactions were not significant (all  *t* < 1.6; *P* > .1), indicating that the variant of the task did not systematically influence the mental rotation process.

### 3.2. Individual Differences: Split-Half Reliability

Across all target stimuli the split-half reliability of the intercepts was “acceptable” to be “good” in both the blocked (reliability = .79) and the mixed variants of the task (reliability = .82). The split-half reliability of the slopes was acceptable for the blocked variant (reliability = .79) but unacceptably low (reliability = .2) for the mixed variant for which the correlation was also nonsignificant (Table 1).

The reliabilities were much lower at the level of individual targets. While the reliabilities for the intercepts were between poor and acceptable (range between .57 and .79) for the blocked variant, the reliabilities in the mixed variant ranged from .34 to .67. None of the slopes had reliabilities larger than .38.

### 3.3. Individual Differences: Test-Retest Reliability

Pooling all targets, the intercepts (Table 2) were significantly correlated at the two-time points. We found slightly higher correlations in the blocked presentation (*r* = .68; *P* < .001) than in mixed presentation (*r* = .51; *P* < .01), suggesting that this variant results in more stable measurements. The slopes showed similarly high levels of retest reliability, again with higher reliabilities for the blocked variants (*r*_tt_ = .69) compared to mixed variants (*r*_tt_ = .55). These numerical differences were, however, not statistically significant.

At the level of individual targets the test-retest reliabilities for the intercepts ranged from .42 to .61 for the blocked presentation and .15 to .58 for the mixed presentation. For the slopes, the only retest reliabilities larger than .50 were found for hand and car targets in the blocked variant presentation and the face targets in the mixed variant.

## 4. Discussion

The aim of the present study was to investigate whether and at what level mental rotation tasks are suitable to assess individual differences. Our study revealed three main results. First, we found acceptable consistency and stability for both the individual intercepts and regression slopes when combining all targets. Second, analyzing subsets of target types separately, we found only acceptable reliabilities for intercepts, while the theoretically more important slopes exhibited very poor split-half and test-retest reliabilities. Third, the blocked variant resulted in a numerically higher consistency and stability and was easier to solve, as indicated by overall shorter reaction times in the group analysis. Before turning to the effects concerning the reliability of mental rotation tasks, we briefly discuss the findings at the group-level.

### 4.1. Group-Level Effects

**Figure 2:**
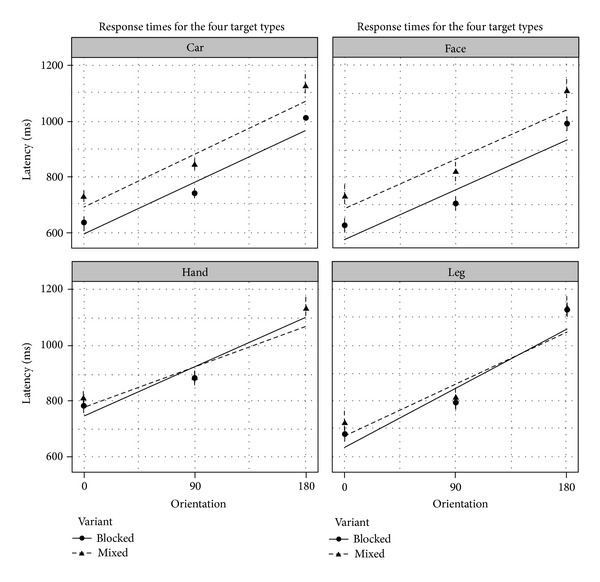
Mean response times in the different conditions (90 deg. medial and lateral rotations were combined). Error bars represent 95% CI. Solid (blocked variant) and broken (mixed variant) lines represent linear fit.

At the group-level we found that most of the effects that replicate earlier work [6, 7] in those rotated targets were associated with about 300 ms slower reaction times (Figure 2). We were also able to systematically compare the different target types that have been trialed before [3, 6, 8, 13]. We found that participants were slower to respond to hand targets compared to the other target types. In an earlier study, Fiorio and colleagues [3] also found the numerically slowest responses for hands but were not able to show reliable differences between different target types. As they included only 24 participants, this discrepancy might be explained by the larger sample size employed here. Together these results also show that participants have no problem solving the more unnatural left-right judgments on faces and cars. Interestingly, the rotation effect was larger for feet. While we did not collect ratings for the individual postures, parsons found that the range of motion was smaller, and the awkwardness ratings were higher for feet to hands [6]. Thus, the larger effect might be due to the fact that mentally simulating these postures was harder for feet compared to the other targets. The factor gender did not emerge as a significant predictor. While mental rotation tasks with three-dimensional objects typically result in large gender effects (see [20] for a meta-analysis) more data are needed to draw firm conclusions on gender differences in the mental rotation of body parts. Finally, we found that participants were overall faster to perform the blocked version of the task, while there was no interaction with the rotation effect. This may indicate that additional processes are related to task switching [21]. Even though the lack of interaction with the rotation effect indicates that this affects only peripheral processes and the analysis at the group level seems to favor the easier blocked variant.

### 4.2. Reliability of Mental Rotation Tasks

Pooling all targets we found only acceptable consistency and stability of the mental rotation indices. Importantly, for the blocked variant of the task this was true both for the intercept and slope parameters. It seems intuitive that the blocked version of the task should give more stable estimates, but our analysis only showed a numerical advantage for this presentation mode rather than true significant differences. However, at the level of individual targets only the intercepts could be assessed with sufficient reliability. Unfortunately, these intercepts mostly reflect peripheral processes and not the theoretically more relevant processes that are related to mental rotation proper [15]. So far, few studies have reported the reliability of mental rotations slope scores. While Kosslyn and colleagues [2] reported very high levels of internal consistency for slope scores, some studies have already highlighted the problems when analyzing slope scores [22, 23]. It is important to note that these low estimates for the internal consistency were found even when using the Spearman-Brown formula to correct for the effect of reducing the number of trials.

These low reliabilities presented here do not indicate that mental rotation tasks cannot be used to assess differences between groups or treatments [4, 5]. But reliabilities of measures set an upper limit for the magnitude of the possible correlation of the two measures. Namely, within classical test theory the correlation coefficient that can be expected between a pair of measures that are perfectly correlated is the square root of the product of the reliabilities of the measures [19]. While the consequences of this relationship have been discussed in the domain of social neuroscience [24] and personality [4, 5], it also has consequences for the interpretation of mental rotation tasks. For example, they may explain the nonsignificant correlations between various tasks of spatial ability [25] and disability scores and performance in an earlier study [3]. As more and more researchers are trying to link aspects of mental rotation tasks to individual differences by using correlation or covariance analysis more attention needs to be paid to these aspects.

As these negative test-retest results for the slopes are in essence nonsignificant correlations, it is important to consider the power of our analysis. As 63 participants completed the retest, the statistical tests for correlations had a very high power. In fact, the power to detect a significant effect (*α* = .05), if the true correlation was at least *r* = .5, was .99 for the test-retest reliability [26]. That is, the chance to miss a substantial effect was smaller than .01. Thus, it is appropriate to conclude that the slope parameters from the mental rotation tasks using individual targets are not suitable to differentiate healthy participants.

### 4.3. Limitations

The study had several limitations that limit the scope of the conclusions. First, the sample was comprised of psychology students only. This might have limited the variance that could be explained, so that the results for the retest reliability might be better in another, for example, patient populations. However, as many experiments are conducted in psychology departments, most samples have a similar composition. We thus believe that our results especially concerning the low retest reliability can be generalized to other relevant studies. Second, we employed a version of mental rotation task with only four different angles of rotation and averaged lateral and medial rotations. While this is at odds with many studies, others used such a task to compare groups. Third, the results of the reliability analysis strongly depend on the number of trials per condition. This is evident by our finding that the intercept and slope parameters were more stable when the target types were pooled. Our data do not allow us to disentangle whether this is due to the number of repetitions or the number of different target types. In any case common designs testing only 8 trials per target and angle of rotation [8, 27] may yield insufficiently reliable estimates for individual differences. The Spearman-Brown formula provides only an estimate for the true reliability that might be achieved with longer tests and may in fact overestimate the reliability. It has been suggested that at least 50 trials per person per condition should be recorded [28]. Given the limited attention span in some diseases or with children [29], it may not be feasible to extend the length of the test. Future studies should try to systematically compare the reliabilities in different number of trials and possible number of angle of rotation to develop evidence-based recommendations, instead of insisting on the maximally possible number of repetitions.

## 5. Conclusions

To sum up, we have presented the first systematic assessment of suitability of mental rotation tasks using body parts as targets to assess individual differences in motor imagery. Across targets both intercepts and slopes could be assessed with acceptable consistency and stability. In contrast, the slopes for individual targets could not be used as measures of individual differences in healthy participants. These low reliabilities might explain null results when trying to relate individuals' performance to disease-related parameters [3]. Researchers in all domains of neuroscience need to be more aware of the importance of reliable measurements as imperfect reliabilities seem to be the rule rather than the exception.

## Figures and Tables

**Table 1:** Split-half reliabilities.

	Intercepts			Slopes		
	Correlation	*P*	Reliability	Correlation	*P*	Reliability
Blocked	.65	<.001	.79	.54	<.001	.79
Hand	.5	<.001	.67	.14	.35	.24
Leg	.44	<.001	.61	−.02	.18	
Face	.43	<.001	.60	.02	.91	.03
Car	.4	<.001	.57	.23	.11	.38
Mixed	.7	<.001	.82	.11	.46	.2
Hand	.44	<.001	.61	−.06	.71	
Leg	.3	.04	.47	.13	.38	.23
Face	.51	<.001	.67	−.08	.60	
Car	.21	.16	.34	.19	.2	.32

Note: reliability for full test adjusted according to Spearman-Brown formula. The formula is not applicable to negative correlations.

**Table 2:** Test-retest reliabilities.

	Intercepts		Slopes	
	Correlation	*P*	Correlation	*P*
Blocked	.68	<.001	.69	<.001
Hand	.61	<.001	.5	<.05
Leg	.53	<.01	.12	.53
Face	.52	<.01	.35	.05
Car	.42	<.05	.57	<.01
Mixed	.51	<.01	.55	<.01
Hand	.39	<.05	.41	<.05
Leg	.45	<.05	.25	.20
Face	.58	<.01	.64	<.001
Car	.15	.426	0	.99
